# Brain Networks of Explicit and Implicit Learning

**DOI:** 10.1371/journal.pone.0042993

**Published:** 2012-08-31

**Authors:** Jing Yang, Ping Li

**Affiliations:** 1 Department of Psychology and Center for Brain, Behavior, and Cognition, Pennsylvania State University, University Park, Pennsylvania, United States of America; 2 National Key Research Center for Linguistics and Applied Linguistics, Guangdong University of Foreign Studies, Guangzhou, China; Institute of Psychology, Chinese Academy of Sciences, China

## Abstract

Are explicit versus implicit learning mechanisms reflected in the brain as distinct neural structures, as previous research indicates, or are they distinguished by brain networks that involve overlapping systems with differential connectivity? In this functional MRI study we examined the neural correlates of explicit and implicit learning of artificial grammar sequences. Using effective connectivity analyses we found that brain networks of different connectivity underlie the two types of learning: while both processes involve activation in a set of cortical and subcortical structures, explicit learners engage a network that uses the insula as a key mediator whereas implicit learners evoke a direct frontal-striatal network. Individual differences in working memory also differentially impact the two types of sequence learning.

## Introduction

Previous research has suggested that explicit learning, a form of conscious, intentional, and declarative process of knowledge acquisition, differs significantly from implicit learning, a form of unconscious, incidental, and procedural knowledge acquisition. These two forms of learning have also been suggested to play distinct roles in natural language acquisition: according to the declarative-procedural model [1], native language learners rely on declarative memory (mostly explicit) for lexical processing and learning but procedural memory (mostly implicit) for grammatical processing and learning; in contrast, second language learners rely on declarative memory to handle both lexical and grammatical processes. Recent neuroimaging data also suggest that distinct and separable cognitive and neural mechanisms underlie explicit and implicit learning: the hippocampus and the temporal-parietal cortex are considered important structures in subserving explicit learning and representation of knowledge [2], [3], whereas a cortical-subcortical circuit (specifically the frontal cortex and the basal ganglia) is believed to mediate implicit learning and memory [4]–[6]. In particular, the medial temporal lobe and the basal ganglia are found to be differentially engaged in explicit versus implicit learning conditions, and as learning progresses, the learner’s dependence on the temporal lobe declines rapidly [7].

While dissociations between implicit and explicit learning have figured prominently in memory research, a growing number of studies have also highlighted how the two types of learning might interact in the learning process, particularly in natural language learning [7]–[12]. Research has indicated that while implicit learners can quickly show competence on original learning materials, they fail in a deeper understanding of the underlying rules; in contrast, explicit learners understand the rules but fail to apply them to novel stimuli. Only when explicit learning and implicit learning occur together can the learner apply the explicit knowledge of the rules to new structures. A given learner may employ both systems by using explicit learning for initial registration of form-meaning associations while using implicit learning for information integration, thus maximally benefitting from both types of learning. However, there might be inherent constraints (e.g., age or learning condition) on the learner’s ability to use one or both types of learning, such that early learning in the natural setting (e.g., first language acquisition by children) depends more heavily on implicit learning, whereas initial learning in the instructed setting (e.g., classroom second language acquisition by adults) relies more strongly on explicit procedures.

The existing literature also suggests that although explicit and implicit learning may be dissociable on both cognitive and neural dimensions, the two types of systems can display in a single individual in terms of resource allocation, information integration, and learning constraints. The precise neurocognitive mechanisms of the two types of learning, however, remain to be determined [13], [14]. In this study, we provide evidence to show that the interaction between, as well as the distinction of, implicit learning and explicit learning mechanisms may be represented by brain networks of differential connectivity, as discussed below. We demonstrate this through learning in a classic paradigm called artificial grammar learning [15].

Recent interests in large-scale brain networks point to a new framework for understanding distinct versus overlapping neural systems, and for identifying the dynamic interactions among neural systems for both normal and impaired brain functions [16]–[18]. This framework views cognitive functions as arising from the interactions between and within distributed brain networks, often constrained by context and mode of learning and processing. With methodological developments in neuroimaging such as structural equation modeling [19] and dynamic causal modeling [20], researchers are now able to do ‘effective connectivity’ analysis to identify the causal interactions among brain regions, rather than just inferring correlational relationships using ‘functional connectivity’ analyses. However, such bidirectional influences in effective connectivity are very complex and may be both activity- and time-dependent. It was not until recently that researchers are able to examine both the activation and connection at a given time (contemporaneously) and their directions of influence across different times (sequentially), through methods such as unified structural equation modeling [21]–[23]. A first objective of the current study is to present evidence based on effective connectivity analyses of functional magnetic resonance imaging (fMRI) data, to show that explicitly and implicitly learned materials involve overlapping neural nodes but differentially weighted brain networks.

A second important objective of this study is to identify whether individual differences in phonological working memory, among other cognitive abilities, would significantly and differentially impact explicit versus implicit learning. It is well known that phonological working memory, the ability to maintain, store, and update phonological sequences, is associated with first and second language learning [24]–[27]. Some studies have suggested that working memory capacity impacts explicit language learning [28], but not implicit learning processes [29], [30]. It has also been claimed that working memory is a strong predictor of second language learning: individuals with higher working memory perform better or more like native speakers than those with lower working memory during the acquisition and processing of lexicon and grammar [25], [31]. Such findings point to the fact that explicit learning rests more heavily on conscious awareness and attentional control, whereas implicit learning does not [32]. However, there is a lack of research in the neural mechanisms underlying the relationship between phonological working memory and implicit and explicit learning conditions. Thus, understanding how phonological working memory differently influences the two types of learning, as well as the neural networks for explicit versus implicit learning, were the research goals of the present study.

**Figure 1:**
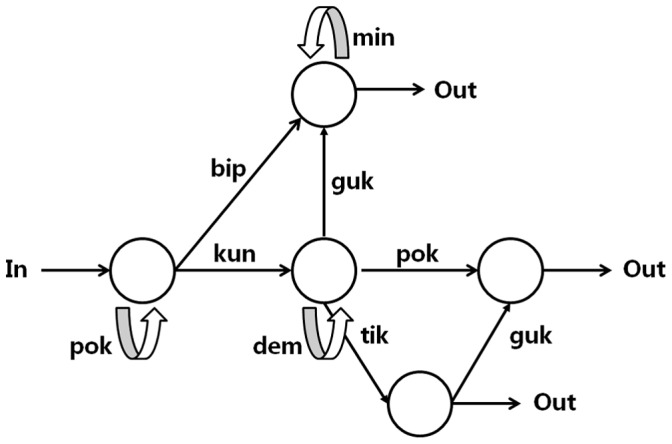
Finite-state grammar used to generate grammatical syllable sequences for explicit and implicit learning. Grammatical syllable sequences are generated by starting at the leftmost ‘In’ state and then following a path of arrows until the rightmost ‘Out’ state is reached. For each arrow traversed, the indicated syllable is added to the syllable sequence. For example, *pok kun dem dem tik* is grammatical, and *pok kun tik guk pok* is ungrammatical.

To answer our research questions, we trained participants with an adapted classic artificial grammar learning (AGL) paradigm [15] that involves syllable sequences generated through a finite-state grammar (**Figure 1**). Finite-state grammars, although limited in their generative capacities [33], are complex systems that can derive sequences through nodes and loops, and the generated sequences either conform to prescribed grammatical rules or do not (see **Materials and Methods** for details). In a typical AGL paradigm, participants are exposed to a subset of letter sequences generated by a finite grammar (the study phase), and then are given a grammaticality judgment test (the test phase) to see whether they can discriminate new grammatical strings from non-grammatical ones. Although participants often show little confidence in their judgments and are unable to consciously report the rules of the grammar, they typically perform above chance on AGL tasks after training. More important for our discussion, learning in the AGL task, although involving artificially generated sequences for training, has been frequently used to infer about natural language acquisition and processing, as have other artificial systems such as Brocanto [34]. Recent neuroimaging data also indicate that structural incongruencies in both artificial grammar learning and natural language learning lead to similar P600 effects, a positive shift in the waveform recorded through event-related potentials (ERP), typically associated with the processing of syntactic or grammatical violations [35].

In our study, the participants underwent a study-test procedure as in the classic AGL task: during the study phase, they were asked to study and memorize sequences of syllables that were grammatical according to the finite-state grammar; during the test phase (retrieval phase), when their brain images were collected, they were told to discriminate whether or not the presented novel sequence conformed to the learned AGL rules (grammaticality judgment; see **Materials and Methods** for details). Forty-three university students, who were performance-matched for IQ, vocabulary, processing speed, and executive control abilities (**Table 1**), were randomly assigned to the explicit versus the implicit learning group and received identical study-test materials with identical procedures, except that at the beginning of the study phase, the explicit group was told of the existence of complex rules underlying the sequences of syllables that the participants were studying, whereas the implicit group was not. We investigated the similarities and differences between the neural networks underlying explicit and implicit learning conditions during the retrieval phase in the fMRI scanning session, and further examined correlations between the participants’ phonological working memory capacity and their neural responses to the two types of learning.

**Table 1:** Summary of participant information and performance scores.

	ExplicitLearners	ImplicitLearners
N	20	23
Age (years)	21.59±2.6	20.39±1.07
Age Range (years)	19.33–30.75	18.17–22.25
Sex (male:female)	9∶11	13∶10
Handedness (maximum:45)	38.4±9.59	41.8±3.33
Nonverbal IQ (%)	51.74±29.68	50.04±22.74
Vocabulary (PPVT-4)	110.72±13.92	109±10.37
Picture Naming (ms)	690.86±126.45	606.29±258.81
Digit Symbol Substitution Test (ms)	1246.75±153.26	1185±170.05
Flanker Task (ms)	51.19±27.17	35.87±34.6
Color-Shape Switch (ms)	202.48±102.26	158.67±105.86
N-back (average accuracy %)	0.77±0.13	0.78±0.14
Letter-Number Sequencing (maximum:8)	5.81±1.11	6.17±1.07
AGL Grammaticality Judgment (%)	58.54±7.5	55.94±8.1

Notes: numbers indicate means and standard deviations (± SD). Measurement scales are in parentheses following the characteristics labels. There are no significant group differences in all measures above, indicating that the two types of learners are highly comparable at a group level.

## Results

There were no significant differences at the group level between explicit and implicit learners in terms of their average age, IQ, vocabulary, processing speed, phonological working memory and other cognitive control abilities (**Table 1**). The explicit learners showed better performance at the IQ and vocabulary tests than the implicit learners, whereas the implicit learners outperformed the explicit learners in processing speed, inhibitory control, and working memory capacity measures. However, these differences were not statistically significant, and were due particularly to outlier performance scores from a few older participants in the explicit group (see our attempt in controlling for age for the partial correlation analysis below).

Response accuracy for grammaticality judgment was 59% (SD = 0.075) and 56% (SD = 0.081) for the explicit group and the implicit group, respectively, consistent with the accuracy range that has been previously reported in the AGL literature [36]. These scores were significantly different from chance (t_19_ = 4.96, explicit group; t_22_ = 3.47, implicit group, both *p*s <.001) but not from each other (t_41_ = −1.07, *p*>.05). Although the explicit and implicit learners did not differ significantly from one another at the group level, some participants from the explicit group noted, in post-training briefings, patterns of repetition and co-occurrence of certain syllables; however, no one was able to articulate the actual finite-state grammar, consistent with findings in the AGL literature.

**Figure 2:**
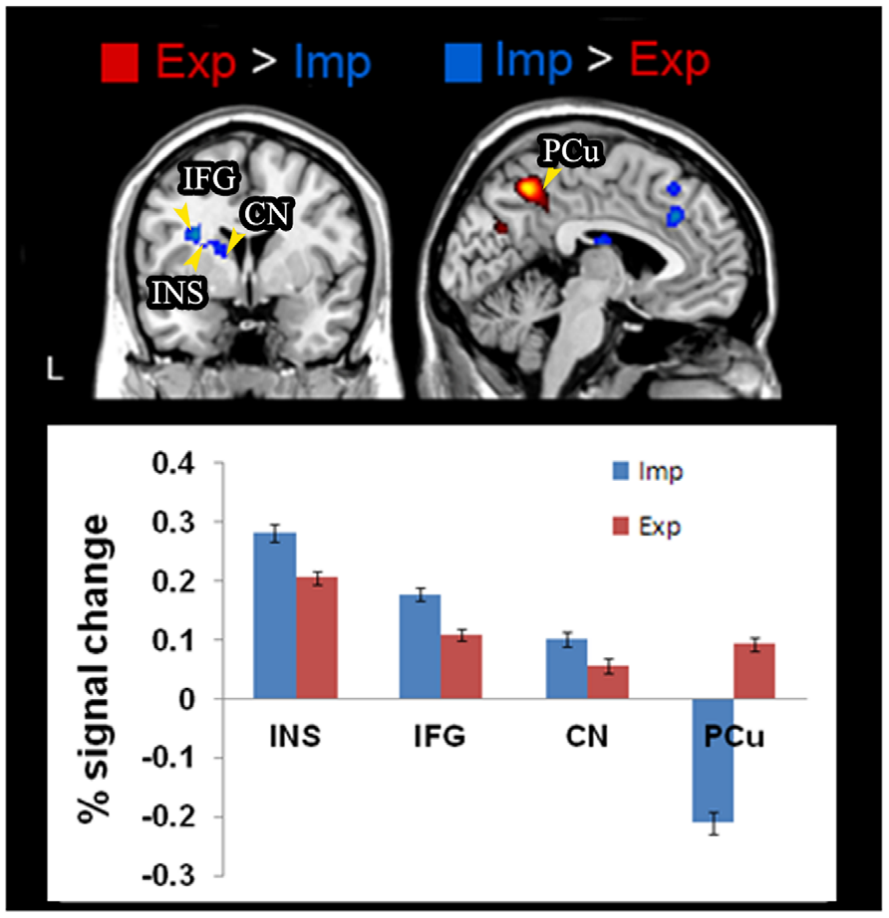
Brain activitions for explicit (Exp) versus implicit (Imp) learners during grammaticality judgment. Four left brain regions showed different blood-oxygenation level dependent (BOLD) signal changes between groups (lower panel): CN = caudate nucleus; IFG = inferior frontal gyrus; INS = insula; PCu = precuneus.

The fMRI data of the grammaticality judgment versus fixation showed common brain activations, regardless of types of learning, in the middle frontal gyrus, supplementary motor areas and occipital regions, and in particular, the hippocampus and the caudate nucleus. Previous research has indicated important hippocampal and caudate functions for motor sequence learning in both explicit and implicit conditions [37], [38]. Direct comparisons between the two groups (**Table 2** **)**, however, revealed significant differences in the precuneus (PCu), with increased activation for explicit learning but decreased activation for implicit learning. Activity in the PCu has been previously suggested to reflect episodic memory retrieval in explicit compared to implicit condition [39]. In contrast to the differences in the PCu, significantly greater activations were observed in the left inferior frontal gyrus (IFG), anterior insula (INS), and caudate nucleus (CN) for the implicit compared to the explicit condition (**Figure 2**). One could claim, on the basis of these direct comparisons, that the contrasting patterns in these brain regions accord with the view that distinct neural mechanisms subserve explicit versus implicit learning, consistent with the extant literature.

**Table 2:** Areas of significant brain activations in the grammaticality judgment task.

Regions activated	L/R	BA	MNI Coordinates			T
			x	y	z	score
**Explicit learners**						
Middle frontal gyrus	L	9	−52	30	32	5.43
	L	10	−38	58	14	5.18
	L	46	−28	52	0	3.99
	R	9	48	38	32	7.35
	R	10	28	54	−8	7.1
Precentral gyrus	L	6	−46	0	34	5.47
Supplementary motor area	R	6	4	22	48	7.94
Insula	L	–	−32	24	−4	5.48
Superior parietal lobule	L	7	−28	−72	50	9.3
Inferior parietal lobule	R	7	38	−54	52	9.45
Precuneus	R	7	8	−72	48	3.05
Inferior occipital gyrus	L	19	−40	−86	−10	9.58
Cuneus	R	17	20	−98	8	12
Hippocampus	L	–	−22	−28	−6	4.19
	R	–	24	−30	−4	4.8
Caudate Nucleus	R	–	18	10	8	2.99
**Implicit learners**						
Middle frontal gyrus	L	46	−40	54	10	6.55
	L	10	−42	48	−14	5.02
	R	9	44	34	26	6.93
	R	6	38	10	54	6.16
Inferior frontal gyrus	L	44	−44	2	30	6.82
Supplementary motor area	R	6	2	20	50	9.7
Insula	L	–	−32	22	−2	7.01
	R	–	32	24	−2	7.37
Cingulate gyrus	L	32	−14	20	18	3.25
Precuneus	L	7	−28	−56	46	7.74
	R	7	28	−28	44	7.69
Inferior occipital gyrus	L	19	−40	−84	−10	8.7
Lingual gyrus	L	18	−16	−92	−4	9.38
	R	17	20	−94	6	11.5
Hippocampus	L	–	−26	−26	−6	4.54
	R	–	26	−26	−6	4
Caudate Nucleus	L	–	−10	−12	22	3.45
**Explicit learners > Implicit learners**						
Superior parietal lobule	L	7	−36	−72	50	2.92
Precuneus(PCu)*	–	7	0	−54	48	3.89
	R	7	14	−30	50	3.18
**Implicit learners > Explicit learners**						
Inferior frontal gyrus (IFG)*	L	44	−36	8	22	2.94
Insula (INS)*	L	–	−30	2	22	3.39
Cingulate gyrus	L	32	−2	30	34	3.43
Caudate Nucleus(CN)*	L	–	−20	4	16	3.03

Notes: L, left hemisphere; R, right hemisphere. All activations reported were thresholded at cluster level p<0.05. * Averaged time course data from all the voxels within a sphere of 6 mm radius in each region of interests (PCu, INS, IFG, CN) were extracted for comparisons of BOLD signal changes with brain networks between the explicit and the implicit group.

**Figure 3:**
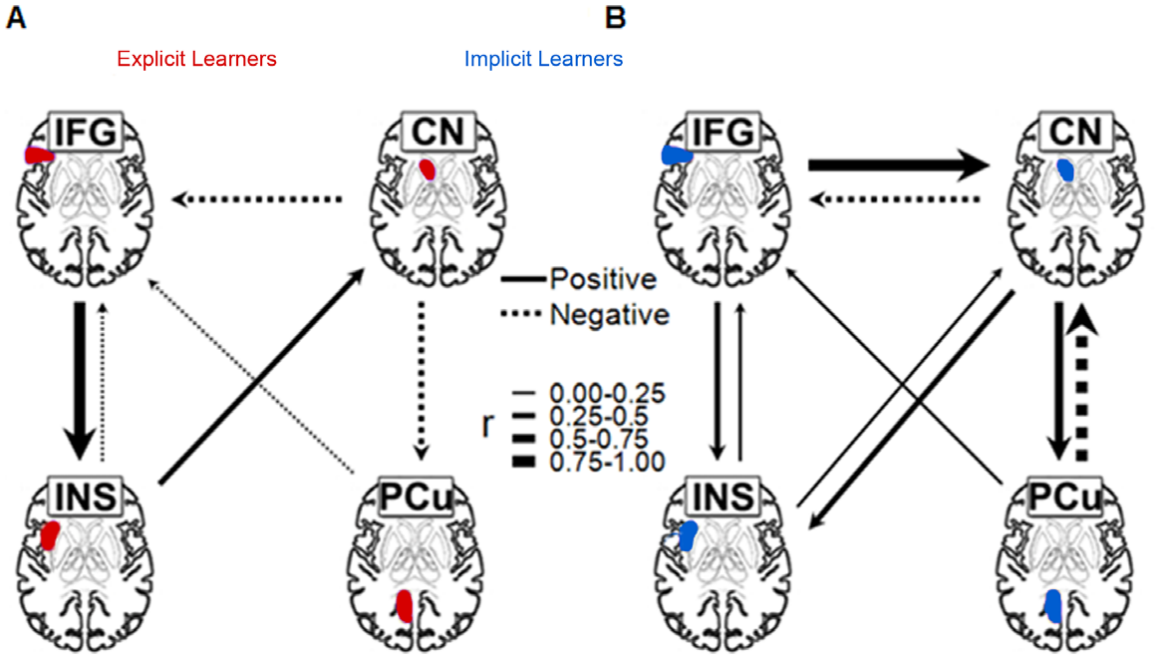
Brain networks for explicit and implicit learners during grammaticality judgment. Positive relationship were denoted by solid lines and negative relationships by dotted lines. See text for further explanation.

We further examined the brain network differences between the two groups by performing effective connectivity analysis on the original time series data from the ROIs (henceforth nodes), using the unified structural equation modeling (uSEM) [21]–[23]. The uSEM has been shown to be a powerful tool for effective connectivity studies, allowing for the identification of both contemporaneous and time-lagged effects and for both data-driven and confirmatory approaches not possible with previous methods (see **Materials and Methods**). **Figure 3** illustrates the contemporaneous connectivity relationships between nodes in the explicit and implicit networks after controlling for time-lagged effects, showing differences in (a) the strength of the connectivity (indicated by the size of the arrows), (b) the directionality of influence from node to node (direction of arrows), and (c) the nature of the connectivity (positive or negative). To see these differences more clearly: first, for explicit learning, IFG positively influences the insula, which then feeds to the caudate (CN). For implicit learning, however, the IFG-to-CN connectivity is direct, without being mediated by the insula. Second, the INS-CN connectivity has different directionality between the two networks, in that the caudate-to-insula influence is dominant for implicit learning whereas the reverse influence is seen for explicit learning. Finally, the caudate also actively interacts with the precuneus for implicit learning, with positive CN-to-PCu influence but a much stronger negative PCu-to-CN influence. For explicit learning, however, there is only negative influence from CN to PCu, and more important, PCu independently and negatively influences IFG, perhaps reflecting the more independent role that PCu plays in the episodic representation of grammatical rules [39].

**Figure 4:**
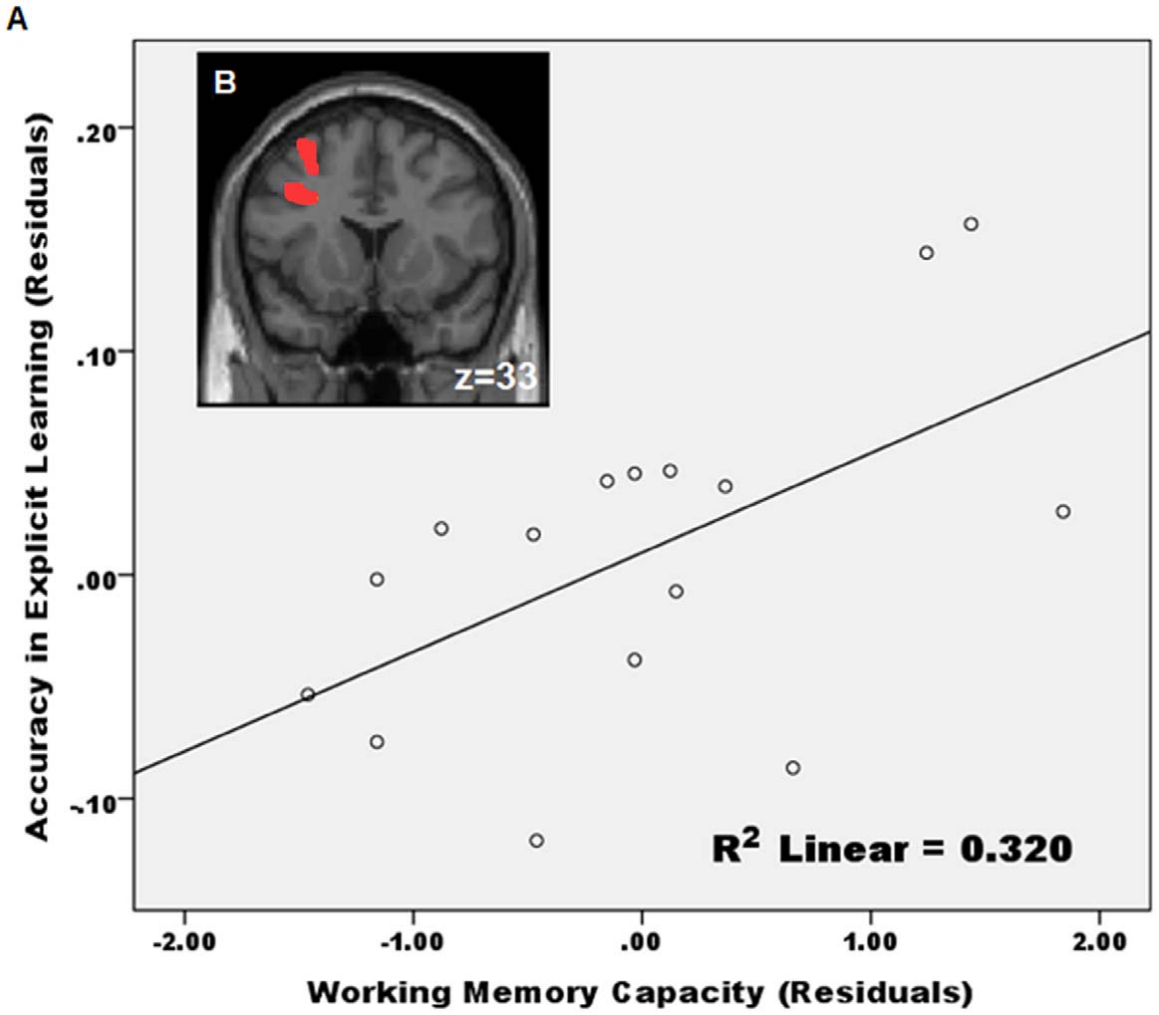
Role of working memory in explicit learning. Working memory capacity in the explicit group showed a positive correlation with behavioral cognitive performance, as seen in (a), and with functional brain activation, as seen in (b), during grammaticality judgment. Residuals were calculated from a partial correlation analysis after controlling for age effects. See text for explanation.

To explore how individual differences in cognitive abilities may impact explicit and implicit learning, we did correlation analyses between grammaticality judgment accuracy in the AGL task and performance scores from the participants’ cognitive tests, which included IQ, vocabulary, processing speed, phonological working memory and inhibitory control. Only the phonological working memory measure showed significant correlation with participants’ grammaticality judgment performance in the AGL task. Given previous findings regarding possible age-related dissociations between explicit and implicit processes [40]–[42], along with age-related variance in our explicit group (see above), we did partial correlation analyses in which age was controlled for. Our analyses indicated that grammaticality judgment accuracy in the explicit, but not the implicit, condition was correlated with participants’ working memory capacity (r = .564, *p*<.05) (**Figure 4a**). Additional correlation analyses showed that the explicit learners’ working memory ability correlated positively with their brain activations in the left dorsolateral prefrontal cortex (DLPFC, BA 9; **Figure 4b**), which included left superior and middle frontal gyrus, whereas the implicit learners’ working memory ability correlated positively with activations in the right DLPFC. The DLPFC, particularly in the left hemisphere, has been consistently implicated as a neural correlate for the central executive function of working memory [24].

## Discussion

The main goal of our research was to examine the neural mechanisms underlying explicit versus implicit grammar learning. There has been a large body of literature that investigates the cognitive mechanisms of the two types of learning, but their neural substrates remain to be understood. In particular, previous cognitive studies have indicated interactions between the two types of learning, whereas neuropsychological and neuroimaging work has focused on their dissociations [6], [39]. In the current study, we sought to explore the connectivity relationships for the relevant brain regions subserving the two types of learning. To achieve this goal, we trained participants with a classic sequence learning task in the artificial grammar learning paradigm, and found that there are overlapping structures with differentially weighted neural networks that underlie performance in the two types of learning. More specifically, our fMRI data indicate that different neural connectivity in the cortical-subcortical structures may be involved in explicit versus implicit sequence learning, with more frontal and parietal structures mediating the attentional and episodic aspects of explicit processes while more direct frontal-striatal relations characterizing the implicit processes.

An interesting though not surprising finding of our study is that while neural patterns of brain response to explicit and implicit learning seem to be clear, there are no significant performance differences between the two groups of learners in general, with respect to both the accuracy of artificial grammar learning and the various cognitive behavioral measures. Several previous studies have shown that explicit versus implicit learning of artificial grammar or second language does not lead to performance differences between groups [43], but in some cases to different neural patterns of response. Morgan-Short, Steinauer, Sanz, and Ullman [44] reported that explicit and implicit second language training led to contrasting patterns in behavioral and neural (ERP) responses. Their participants, regardless of second language proficiency, did not differ in behavioral performance. However, their implicit learning condition elicited a typical brain activation pattern comparable to pattern in native speakers whereas the explicit learning condition did not. Other studies using the ERP method have also demonstrated cases where ERP measures and behavioral measures are inconsistent: where the behavioral measures show no differences, the more sensitive ERP measures can pick up the learning or performance differences (see [45] for review). Thus, the lack of behavioral differences in our study does not indicate the lack of differences between the two types of learning, as the learning differences may be reflected only in neural response patterns.

Our effective connectivity analyses based on the fMRI data yielded three main findings. First, across both learning groups, the IFG served as the ‘hub’ of neural activities, exerting strong top-down influences on other nodes. For explicit learning, there is only one top-down path from IFG to INS; for implicit learning, however, the IFG-to-CN connectivity is direct and parallel to IFG-INS-CN, without being mediated by the insula. This difference between the two types of learning is highly significant, because the insula has been previously identified as a core structure of the Salience Network, which plays a central role in mediating access to attention and memory and switching between endogenously generated mental states (Default Mode Network) and exogenously generated cognitive activities (Cognitive Control Network) – these three networks are considered the core brain networks of cognition, according to Bressler and Menon [16]. The insula as a mediator between IFG and CN in the explicit but not the implicit network suggests higher-order attentional/intentional processing dedicated to explicit learning, as well as a possibly stronger engagement of articulatory planning and control processes involved. Second, the INS-CN connectivity is bidirectional in implicit learning, instead of one way as in explicit learning. Given the significant role that caudate plays in implicit learning [46]–[48], this pattern suggests that the implicit learning network recruits the caudate as a secondary hub of neural activity while it places insula as subsidiary: rather than responding to salient stimuli automatically as in explicit learning, the insula becomes activated as a result of implicit sequence learning. Finally, the connectivity between caudate and precuneus are different between explicit and implicit learning. For explicit learning, PCu passively receives information, not directly from the frontal lobe or the insula, but from the caudate. In contrast, PCu actively influences caudate and positively influences IFG in implicit learning. However, the relationship between PCu’s regional activation level and its neural connectivity with other brain areas needs further investigation. Taken together, our connectivity analysis suggests that underlying similar behavioral performance, explicit and implicit learning conditions rely on different interactions between brain regions such as the inferior frontal gyrus, the caudate, and the precunues.

Our study additionally indicates that individual differences in working memory may impact success in sequence learning, and the differently lateralized neural structures may be implicated in the deployment of working memory depending on the type of learning. The left dorsolateral prefrontal cortex, as shown in our results, may serve as an important neural marker for successful sequence learning in the explicit condition. The role of DLPFC in working memory and its relationship to the two types of learning suggest that we need to further consider individual differences in learning and their neural correlates. For now, the fMRI data and the brain networks analysis presented here have allowed for a new understanding of the common versus distinct neural substrates underlying explicit and implicit learning.

## Materials and Methods

### Ethics Statement

The study was approved by the Social, Life, and Engineering Sciences Imaging Center and the Institutional Review Board of the Pennsylvania State University. Informed written consents were obtained from all participants before the experiment.

### Participants

Forty-three native English speakers from the Pennsylvania State University participated in the experiment and received payment for their participation. All participants had normal or corrected-to-normal vision, and reported no physical or mental disabilities. They were all right-handed as judged by the handedness questionnaire of Snyder and Harris [49].

### Verbal and Nonverbal Tasks

All the participants received a battery of computerized tests on nonverbal IQ, vocabulary, phonological working memory, processing speed, and cognitive control abilities before they began training on the artificial grammar learning (AGL) task. The test results indicated that participants did not differ significantly on cognitive and linguistic abilities (**Table 1**). Only the phonological working memory results were reliably correlated with participants’ AGL performance (see **Results**).

#### Nonverbal intelligence task

Raven’s Standard Progressive Matrix sets [50] were used to measure nonverbal intelligence. The matrices were scanned and were presented to the participants via the E-prime software, version 2.0 (Psychological Software Tools, Inc., http://www.pstnet.com/eprime.cfm).

#### Vocabulary task

The Peabody Picture Vocabulary Test (PPVT, 4th ed.) [51] was used in the current study to measure the participants’ receptive vocabulary in English.

#### Phonological working memory task

The Letter-Number Sequencing task, adapted from that of WMS-III [52], was used to measure phonological working memory capacity. The participants heard a series of alternating numbers and letters at the rate of about one per second, and were asked to report first the numbers in ascending numerical order and then the letters in alphabetical order. The task began with one number and one letter and continued to a maximum of four numbers and four letters alternating in the sequence.

#### Processing speed tasks

The Digit Symbol Substitution task and the Picture Naming task were used to measure nonlinguistic and linguistic processing speed, respectively. The former task is a computerized version of the Digit Symbol-Coding subtest from WAIS-III while the latter task asks participants to name visually presented familiar pictures. The pictures, corresponding to high-frequency words in English, were selected from the UCSD International Picture Naming Project [53].

#### Cognitive control tasks

The Flanker task, the Color-Shape Switching task, and the N-back task were used to measure inhibition, shifting, and updating components of cognitive control abilities, respectively [54]. In the Flanker task [55], participants were asked to press the left or the right button to indicate the direction of a red arrow, flanked by other arrows in a sequence. The directions of the target arrow and the flanker arrows were either the same or different. In the Color-Shape Switching task [56], participants were asked to judge either the shape or the color of two targets (triangles or squares in either red or blue) depending on the task cue. The cue for color judgment was a painting palette and the cue for shape judgment was the same palette in black and white. In the N-back working memory task [54], participants were instructed to press a response button as quickly and as accurately as possible whenever the currently presented letter was identical to a pre-specified letter in a given series of letters (0-back) or the one that occurred 1 or 2 items before its onset, in 1-back or 2-back task, respectively.

### Artificial Grammar Learning

Grammatical sequences were generated from a Markov-chain finite-state grammar [15]. All grammatical sequences were formed by following the path of arrows from ‘In’ to any terminal of ‘Out’ in the diagram (**Figure 1**). For each arrow traversed in the path, the indicated syllable was added to the sequence until ‘Out’ was reached. Ungrammatical sequences used in the test phase did not follow the In-to-Out paths or skipped a necessary syllable. The length of the grammatical and ungrammatical sequences varied from two to five syllables.

According to the classic artificial grammar learning (AGL) paradigm, participants undergo a study phase and a test phase. In the current experiment, the participants were trained on 22 grammatical sequences in the study phase. Each syllable in the sequence was presented visually for 800 ms, with an inter-stimulus-interval of 200 ms between one syllable and the next. The E-prime 2.0 software was used for stimulus presentation and data registration. At the end of the presentation of a sequence, participants were asked to recall the sequence by typing the syllables in the correct order of the sequence. When finished typing, they were shown the correct sequence on the computer screen as a feedback, regardless of whether the sequence they typed was correct or not. Participants received the 22 sequences in increasing length: they were first presented with the two-syllable sequences, which gradually increased in length, until all the five-syllable sequences were presented. This presentation was repeated twice for each participant so that the participant received a total of 66 sequences. All participants were told to memorize the sequences for a memory task after the training. The explicit learning group and the implicit learning group received the same study and test materials, except that the explicit learning group was told at the beginning of the study phase that there were complex rules underlying the sequences of syllables just as grammar rules underlying sentences. In the test phase, both groups of participants were asked to make a grammaticality judgment to novel sequences that either followed or did not follow the rules of the learned AGL sequences. These novel sequences were matched in syllable complexity and sequence length to the original material used in the study phase.

### fMRI Procedure

The study phase of the AGL was conducted outside the fMRI scanner whereas the test phase inside the scanner. During the test phase in the scanner, participants made grammaticality judgments to the new syllable sequences, with each sequence presented in its entirety for 3000 ms, preceded by a fixation of 500 ms and followed by an inter-stimulus-interval of 500 ms. There were eight blocks for the grammaticality judgment and each block consisted of a 2-sec instruction period followed by six trials (26 sec in total). Participants indicated a positive response (“yes – sequence seen was grammatical”) by pressing a response key with their right (dominant) index finger and a negative response (“no – sequence seen was ungrammatical”) by pressing a response key with their left index finger. All the task blocks were interleaved with resting periods (16 s), during which time participants were asked to look at a fixation cross.

### MRI Acquisition

MRI images were acquired on a Siemens Magnetom Trio 3T MRI scanner at the Social, Life, and Engineering Sciences Imaging Center, Pennsylvania State University, using a T2*-weighted gradient-echo EPI sequence (TE = 30 ms; TR = 2 s; flip angle = 90°; matrix size = 80×80 mm; FOV = 240 mm). Participants lay supine in the scanner with plastic ear canal molds, and viewed the visual stimuli via a mirror apparatus mounted on the head coil, while their heads were immobilized with cushions. Functional images were reconstructed from 34 axial slices, with the thickness of each slice being 4 mm. For each run, the functional scanning was always preceded by 6 s of dummy scans to ensure tissue steady-state magnetization. High-resolution (1×1×1 mm^3^) anatomical images were acquired using a T1-weighted, 3D inversion-recovery gradient-echo (MP-RAGE) sequence.

### fMRI Data Analysis and Connectivity Analysis

The data were preprocessed and analyzed with the Statistical Parametric Mapping software (SPM8; Wellcome Trust Centre for Neuroimaging, University College London, http://www.fil.ion.ucl.ac.uk/spm). The first three scans (dummy scans) of each participant’s data set were discarded to allow for T1 equilibration. The remaining volumes were realigned to the first volume, normalized to the EPI template in SPM8 based on the Montreal Neurological Institute (MNI) stereotactic space, and then resampled into 2×2×2-mm cubic voxels. Finally, the images were spatially smoothed with an isotropic Gaussian kernel (9 mm full width at half-maximum [FWHM]). Differences in the activation maps between the two groups were tested by an independent-samples t-test in SPM8 using contrast images for the grammaticality judgment task versus the baseline condition (fixation). Statistical maps were thresholded at p<.05 familywise error rate (FWER) corrected for multiple comparisons.

To arrive at connectivity models for the two groups, original time series from four regions of interests (inferior frontal gyrus, BA 44; insula; caudate nucleus; precuneus, BA7) in the left hemisphere were extracted using the Marsbar toolbox [57]. These regions were chosen based on previous findings on neural correlates for explicit and implicit learning, and on the peak clusters from our group contrasts (**Table 2**). Averaged time course data of all the voxels within a sphere (6 mm radius) in each region of interest (ROI) were extracted for each individual imaging dataset and sorted by experimental conditions (e.g., artificial grammar judgment versus fixation). The averaged time course signals across all trials were converted to percentage signal changes (PSC) using the formula (signal – baseline)/baseline × 100 for each time point, where the baseline constant was the mean signal of the fixation periods. The averaged PSC value for each task was considered as a representative activation level of each ROI for each participant and the group differences in PSC values were illustrated in **Figure 2**.

Connectivity between these regions was determined by using the unified structural equation model (uSEM) [23] with automatic search procedure [21]. The majority of current statistical techniques (e.g., structural equation modeling, vector autoregression) for assessing effective connectivity in functional MRI data identify either contemporaneous or time-lagged effects, which is problematic since both must be considered simultaneously for unbiased parameter estimation. Dynamic Causal Modeling allows for both effects to be built into the model, but it requires *a priori* specification of connections among ROIs with a confirmatory approach [20]. The uSEM method specifies, estimates, and compares all possible models (or at least a large selection of plausible models); in so doing it obtains both contemporaneous and time-lagged effects for all nodes simultaneously within the best-fitting model in an exploratory fashion, thus allowing for automatically search for an optimal model without prior specification of contemporaneous and sequential relationships among ROIs. The uSEM model can be captured in the following equation [23], where η indicates the ROI time series (with time indicated by t, t+1, etc.), A the contemporaneous relations among ROIs, Φ the lagged associations, and ζ the error residual assumed to be a white noise.
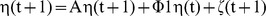



Covariance matrices were created for each of the individual ROI time series for our AGL task. Individual matrices were then pooled to create a group matrix representing correlations between ROIs. The group correlation matrices used for connectivity analysis were 8×8, which included four ROI time series at time t and the same four ROI time series at the next time t +1 (lagged series). The automatic uSEM search procedure was applied to each group matrix. Model fit parameters found to demonstrate reliability in simulation studies [21], [22] were chosen *a priori* so that all the following criteria were satisfied in the final model: root mean squared error of approximation (RMSEA) <0.05; standardized root mean squared residual (Standardized RMR) <0.05; non-normed fit index (NNFI) >0.95; and the comparative fit index (CFI) >0.95. This procedure offers a conservative estimate of connection β values that are less sensitive to sample size and number of parameters [58]. **Figure 3** illustrates the unbiased interactions between nodes of the networks after controlling for the lagged relationship.
